# Redox Signaling Biomarker and Transcription Factor Assessments Are Important to Evaluate Myocardial Protection Status Through Different Cardioplegias

**DOI:** 10.21470/1678-9741-2024-0315

**Published:** 2025-08-04

**Authors:** Tamer Cebe, Seydanur Turgut, Fatih Kızılyel, Erdem Atasever, Onur Sokullu, Bülend Ketenci, Gülnur Andican, Ufuk Çakatay

**Affiliations:** 1 Department of Cardiovascular Surgery, Dr. Siyami Ersek Chest, Heart, and Vascular Surgery Training and Research Hospital, Istanbul, Turkiye; 2 Department of Medical Biochemistry, Cerrahpaşa Faculty of Medicine, Istanbul University-Cerrahpaşa, Istanbul, Turkiye

**Keywords:** Cardiac Surgical Procedures, Coronary Arterty Bypass, Induced Heart Arrest, Biomarkers, Oxidation-Reduction.

## Abstract

**Introduction:**

Cardioplegias are routinely used in cardiac surgery to protect the heart from ischemia-reperfusion injury. The choice of cardioplegia depends on the surgeon's clinical expertise. No clear data demonstrate the redox-protective superiority of one cardioplegia over another. We aim to evaluate redox status and signaling assessments in coronary sinus blood samples for the different cardioplegias.

**Methods:**

Our study included patients undergoing coronary artery bypass and isolated valve surgery. We compared blood and del Nido cardioplegia solutions. During the preoperative period, blood samples were collected from the coronary sinus both preand post-aortic cross-clamping. We also assessed redox system biomarkers and transcription factors related to the antioxidant system using spectrophotometric and immunochemical methods.

**Results:**

In valve patient groups that received both cardioplegia solutions, post-cross-clamping protein carbonyl levels were significantly lower compared to pre-cross-clamping values. For the levels of antioxidant system parameters, except for catalase and superoxide dismutase, no significant difference was observed for del Nido cardioplegia. Increased antioxidant enzyme levels highlight the importance of these enzymes in eliminating the higher hydroperoxide load. Regulatory proteins involved in redox signaling did not show significant variations except for Kelch-like ECH-associated protein 1 and peroxisome proliferator-activated receptor-gamma coactivator-1 alpha for cardioplegias.

**Conclusion:**

Current results indicate that del Nido cardioplegia effectively protects myocardial redox status. Given these findings, despite concerns regarding its use in clinical practice, particularly in valve surgery compared to coronary artery bypass surgery, del Nido cardioplegia may provide effective myocardial protection in both coronary artery bypass and heart valve surgeries.

## INTRODUCTION

**Table:** 

Abbreviations, Acronyms & Symbols				
AOPP	= Advanced oxidation protein products		Keap1	= Kelch-like ECH-associated protein 1
AUC	= Area under the curve		L-OOH	= Lipid hydroperoxides
AVR	= Aortic valve replacement		LDL	= Low-density lipoprotein
BT-LAB	= Bioassay Technology Laboratory		MnSOD	= Manganese superoxide dismutase
CAT	= Catalase		Nrf2	= Nuclear factor erythroid 2-related factor 2
CPB	= Cardiopulmonary bypass		PCO	= Protein carbonyl group
CRP DNPH	= C-reactive protein = 2,4-dinitrophenylhydrazine		PGC-1α	= Peroxisome proliferator-activated receptor-gamma coactivator-1 alpha
EF	= Ejection fraction		ROC	= Receiver operating characteristic
ELISA	= Enzyme-linked immunosorbent assay		ROS	= Reactive oxygen species
GPx	= Glutathione peroxidase		SD	= Standard deviation
IL	= Interleukin			

Cardioplegias are routinely used in cardiac surgery to protect the heart from ischemia. Choice of the cardioplegia depends on the surgeon's clinical experience and preference. Various cardioplegia solutions (such as Bretschneider, del Nido, blood cardioplegia, crystalloid cardioplegia, St. Thomas) are widely used in clinical practice^[1]^. No clear scientific data in the current literature demonstrates the superiority of one cardioplegia over the others^[2]^. In this study, we preferred the blood and del Nido cardioplegia solutions, which are commonly used in our clinic. Blood cardioplegia is an autologous cardioplegia that includes physiological buffer systems, allowing for heart nourishment and containing native antioxidant substances. However, the need for repeated doses every 20 minutes after the initial application creates a disadvantage in terms of surgical comfort^[3]^. On the other hand, del Nido cardioplegia is preferred by surgeons in complex cases due to its long application intervals. The adequacy of a single dose for up to 90 minutes after the initial application enhances surgical comfort by allowing the procedure to be carried out meticulously without interruptions and by maintaining the surgical position. However, the content being predominantly electrolyte-based, containing a 1:4 ratio of autologous blood, and the extended time of a single dose are disadvantages compared to blood cardioplegia in terms of heart nourishment^[1]^. Hamad et al.^[3]^ reported that del Nido and blood cardioplegia solutions offer equivalent safety in combined adult valve surgery.

Reactive oxygen species (ROS) and reactive nitrogen species can lead to oxidative damage of endothelial cells but also adverse redox signaling at the level of kinases, calcium handling, inflammation, epigenetic control, circadian clock, and proteasomal system. Pharmacological interventions to regulate redox imbalances lead to a normalization of these redox biomarkers as well as functional parameters^[4]^. Myocardial protective effects of cardioplegias on myocardial redox homeostasis are among the current research topics. Ischemia and reperfusion injury can be observed in the myocardial tissues of patients undergoing coronary artery bypass graft surgery and isolated valve replacement surgery. Blood drawn from the coronary sinus provides information on the specific pathophysiology of heart conditions^[5]^. Comparative research examining the effects of blood and del Nido cardioplegias on coronary sinus redox status is not currently available in the literature.

Protein carbonyl groups (PCO) and advanced oxidation protein products (AOPP) are widely used biomarkers of cardiovascular redox status^[6,7]^. Transcription factors such as nuclear factor erythroid 2-related factor 2 (Nrf2) and peroxisome proliferator-activated receptor-gamma coactivator-1α (PGC-1α) play important roles in controlling physiological functions through redox regulation mechanisms in cardiovascular tissue^[8,9]^. Nrf2 stimulates the expression of enzymatic antioxidant systems, transcription factors, redox signaling proteins, and cytoprotective detoxifying enzymes in response to oxidative stress during atherosclerosis-related cardiovascular diseases^[10]^. It plays a protective role against oxidative stress in cardiopulmonary bypass (CPB). Nrf2 induces the activation of structural genes encoding enzymes such as NAD(P)H dehydrogenase (quinone 1), cytosolic and manganese superoxide dismutase (MnSOD), catalase (CAT), and glutathione peroxidases (GPx) in redox signaling pathways through the antioxidant response element regulator nucleotide sequence. Moreover, the Nrf2-Kelch-like ECH-associated protein 1 (Keap1) relationship is a crucial event for regulating redox homeostasis in myocardial tissue. Nrf2 is sensitive to changes in redox status and interacts with Keap1, a protein that regulates redox homeostasis in myocardial tissue^[10]^. PGC-1α, on the other hand, acts as a coactivator activating transcription factors in different metabolic pathways. It promotes mitochondrial biogenesis and oxidative metabolism^[9]^.

Understanding the relationship between redox biomarkers and transcription factors in coronary sinus samples helps evaluate cardioplegia effectiveness in open heart surgery. Our study aims to contribute to the development of strategies to minimize oxidative damage associated with cardiac surgery by investigating the effects of blood cardioplegia and del Nido cardioplegia solutions on myocardial redox homeostasis during the postoperative periods using redox biomarkers and antioxidant system transcription factors. We intend to compare these redox effects with elective isolated valve replacement surgery patients to minimize oxidative damage associated with cardiac surgery.

## METHODS

### Study Design

Cases included in our study were selected from patients who underwent elective coronary artery bypass grafting (n = 28) and those who underwent elective isolated valve replacement surgery (mitral valve replacement: three; aortic valve replacement [AVR]: 23) without coronary artery disease (n = 26) at Dr. Siyami Ersek Chest, Heart, and Vascular Surgery Training and Research Hospital between January 2023 and July 2023. The sample size was calculated to be a minimum of 24 people using a 95% confidence interval and a 0.95 power ratio. The analysis was performed in the G-Power statistical analysis program, version 3.1.9. Inclusion criteria for the study were: i. patients aged between 18 and 75 years; ii. elective coronary artery bypass surgery; iii. patients without coronary artery disease undergoing elective valve replacement surgery; iv. body mass index between 18.5 and 30 kg/m^2^. Exclusion criteria for the study were: i. patients younger than 18 or older than 75 years; ii. patients requiring combined surgery with both coronary artery bypass and valve surgery. iii; obese patients with a body mass index > 30 kg/m^2^; iv. patients who underwent emergency coronary artery bypass surgery.

### Patients’ Data

Data for 28 patients who underwent coronary artery bypass graft surgery and 26 patients who underwent isolated valve surgery are presented in Table 1. There was no significant difference among the data of coronary patients and their respective valve controls concerning age, sex, and preoperative, operative, and routine biochemistry data. The insignificant demographic/laboratory values of Table 1 indicate that our cardioplegia-related oxidation findings are independent of the aforementioned conditions.

**Table 1:** Patients’ data.

	Coronary patient group (n = 28)	Valve patient group (n = 26)	*P*-value
Sex			0.146
Male	24 (85.7%)	18 (69.2%)	
Female	4 (14.3%)	8 (30.8%)	
Age (years)	61.43 ± 6.292	58.15 ± 11.712	0.275
Total cholesterol (mg/dL)	191.14 ± 44.51	192.00 ± 36.58	0.910
High-density lipoprotein cholesterol (mg/dL)	37.36 ± 7.631	39.96 ± 6.384	0.165
Low-density lipoprotein cholesterol (mg/dL)	119.32 ± 40.35	109.12 ± 35.64	0.416
C-reactive protein (mg/L)	94.68 ± 25.56	88.88 ± 33.40	0.153
Cross-clamping time	73.43 ± 19.323	84.23 ± 22.079	0.083
CPB time	109.25 ± 24.517	119.85 ± 28.335	0.185
Preoperative EF%	53.21 ± 8.630	54.23 ± 7.442	0.676
Weaning CPB time	35.82 ± 13.955	35.62 ± 12.949	0.910

*P* > 0.05 for all parameters

CPB=cardiopulmonary bypass; EF=ejection fraction

### Ethical Statement

The study protocol was approved by the local Ethics Committee of the Haydarpaşa Numune Training and Research Hospital (file number: HNEAH-KAEK 2022/KK/183-3914), and it was conducted following the Declaration of Helsinki.

### Surgical Procedure

All cases were performed with full sternotomy, and CPB was performed in mild hypothermia at 32°C. The initial dose of blood cardioplegia solution (4: blood, 1: crystalloid) was administered in 1000 mL antegrade, followed by continuous or every 15 to 20 minutes in both retrograde and antegrade fashion. The del Nido cardioplegia solution was prepared by adding the following components to 1000 cc of Plasma-Lyte A™ solution and preserved at 4°C: 26 mEq of potassium chloride, 16 ml of 20% mannitol, 4 ml of magnesium sulfate (50%), 13 mL of sodium bicarbonate (8.4%), and 6.5 ml of 2% lidocaine. del Nido cardioplegia was administered in 1059 mL single dose antegrade. In patients undergoing coronary artery bypass grafting with del Nido cardioplegia, anastomotic control was done with 5 - 10 cc of del Nido solution. After distal coronary anastomoses were performed, proximal anastomoses were done to the aorta cross-clamping.

Cardioplegic solutions were administered as standard antegrade in valve patients without coronary artery disease. While del Nido was given as a single dose (1000 mL), blood cardioplegia was administered with an initial dose of 1000 mL followed by continuous or every 15 to 20 minutes in both retrograde and antegrade fashion. In surgeries where we anticipated the cross-clamping time would exceed 60 minutes, we administered the second dose of del Nido cardioplegia at the 60^th^ minute. In individual practice, most surgeons will re-dose del Nido cardioplegia between 60 and 90 minutes of ischemic time, with a dose ranging from 250 to 500 mL chosen based on the anticipated remaining length of cross-clamping time^[11]^. Transesophageal echocardiography was used to determine perioperative gradients, assess valve incompetence, and evaluate the presence of paravalvular leaks. Graft flows were evaluated by Doppler ultrasonography.

### Sample Collection

#### Coronary Sinus Blood Sampling

Blood samples were drawn from the coronary sinus pre-CBP and post-cross-clamping removal. The CPB began with standard procedures, utilizing aortic and venous cannulation. Before the CPB, while the heart beats, the retrograde catheter was precisely inserted into the right atrium through a small incision with a purse string suture. Simultaneously, the surgeon exposed the atrioventricular groove by retracting the heart cephalad. Using the right hand, the gently curved retrograde catheter engaged the coronary sinus. The catheter's placement was confirmed using transesophageal echocardiography. After securing the catheter with an inflated balloon, any air was expelled, and a 2 cc sample was stored for analysis. Coronary sinus samples after cross-clamping were collected at four minutes post cross-clamping removal^[12]^.

### Sample Preparation and Storage

Serum samples were obtained by centrifuging blood collection tubes containing coronary blood samples without additives for 15 minutes at 3000 rpm and 4°C. Serum aliquots were frozen and kept at -80°C until they were tested to analyze transcription factors and redox biomarkers.

### Analytical Assays

We investigated the effects of CPB on myocardial redox homeostasis by examining blood redox biomarkers and antioxidant system transcription factors using spectrophotometric and immunochemical methods in the preoperative and postoperative periods.

### Protein Oxidation Biomarkers

#### Protein Carbonyl Groups

Protein carbonylation was assayed with the spectrophotometric method based on a chemical derivatization step of carbonyl groups with 2,4-dinitrophenylhydrazine (DNPH)^[13]^. Unreacted DNPH and lipid remnants were removed with ethanol:ethyl acetate mixture. DNPH-modified PCO content was calculated from maximum absorption (360 nm) using the molar extinction coefficient (ɛ = 22000 L/mol.cm). The PCO concentration was expressed as nmol/mg protein.

#### Advanced Oxidation Protein Products

Assessment of AOPP levels was realized according to the previously described method of Hanasand et al. (2012)^[14]^. Coronary sinus blood samples were diluted with citric acid and then reacted with the potassium iodide reagent. Reaction mixture's absorbance was quickly measured at 340 nm against the reagent blank. The standard curve was used to express the AOPP concentrations as micromoles per liter of chloramine-T equivalents.

### Lipid Peroxidation Biomarkers

#### Lipid Hydroperoxides

The ferric-xylenol orange method for assessment of lipid hydroperoxides (L-OOH) is based on an assay that employs the reduction of peroxides in an acidic milieu by Fe^2+^ and formation of the colored ferric-xylenol orange complex with an absorbance peak at 560 nm^[15]^. Lipid peroxidation of coronary sinus blood samples was assessed with a calibration curve for peroxide value using xylenol orange and Fe^3+^ chloride.

### Antioxidant Capacity Markers

#### Manganese Superoxide Dismutase Expression

MnSOD expression was assessed using a sandwich enzyme-linked immunosorbent assay (ELISA) kit (Bioassay Technology Laboratory [BT-LAB], Shanghai Korain Biotech Co, Shanghai, China). MnSOD concentrations of the coronary sinus blood samples were expressed as ng/mL.

#### Glutathione Peroxidase Activity

GPx activity was analyzed using a GPx assay kit (BT-LAB, Shanghai Korain Biotech Co, Shanghai, China), and the GPx activities of the coronary sinus blood samples were expressed as U/mg protein.

#### Glutathione Peroxidase Expression Level

GPx expression was analyzed using a sandwich ELISA kit (BT-LAB, Shanghai Korain Biotech Co, Shanghai, China), and the GPx concentrations of the coronary sinus blood samples were expressed as µU/mL. This commercial assay kit has a limit of quantification of 1.44 µU/mL According to the kit manufacturer's methodology, the intra-assay coefficient of variability (accuracy within an assay; known concentrations were tested on one plate to estimate the intra-assay precision by the manufacturer) was 3.2.

#### Catalase Activity

CAT activity was measured according to the method described by Aebi (1984)^[16]^. The decrease in absorbance at 240 nm is a direct indicator of H2O2 breakdown. The CAT activity is measured as the difference in absorbance per unit time.

#### Catalase Expression

CAT expression was analyzed using a sandwich ELISA kit (BT-LAB, Shanghai Korain Biotech Co, Shanghai, China), and the CAT concentrations of the coronary sinus blood samples were expressed as KU/L. This commercial test kit's limit of quantification was 1.12 KU/L. According to the kit manufacturer's methodology, the intra-assay coefficient of variability was 3.4.

### Transcription Factors

#### Nuclear Factor Erythroid 2-Related Factor 2

Nrf2 was analyzed using a sandwich ELISA kit (BT-LAB, Shanghai Korain Biotech Co, Shanghai, China), and the serum Nrf2 levels of the samples were expressed as ng/L. This test kit has a sensitivity of 0.11 ng/mL According to the kit manufacturer's methodology, the intra-assay coefficient of variability was 6.2.

#### Kelch-Like ECH-Associated Protein 1

Keap1 was analyzed using a sandwich ELISA kit (BT-LAB, Shanghai Korain Biotech Co, Shanghai, China), and the serum Keap1 levels of the samples were expressed as ng/L. This test kit has a sensitivity of 7.87 ng/L. According to the kit manufacturer's methodology, the intra-assay coefficient of variability was 3.7.

#### Peroxisome Proliferator-Activated Receptor-Gamma Coactivator-1 Alpha

A sandwich ELISA kit (BT-LAB; Shanghai Korain Biotech Co, Shanghai, China) was used to analyze PGC-1α, and the samples' serum PGC-1α levels were reported as ng/mL. The sensitivity of this assay kit was 0.021 ng/mL. According to the kit manufacturer's procedure, the intra-assay coefficient of variability was 4.4.

### Routine Clinical Chemistry Parameters

Routine clinical chemistry parameters were determined on the Cobas Integra autoanalyzer (Roche Diagnostics, Switzerland).

### Statistical Analysis

The sample size for comparing the means of the two groups was calculated using the G-Power 3.1.9 program^[17]^. Statistical analysis of the data was conducted using the IBM Corp. Released 2023, IBM SPSS Statistics for Windows, version 29.0, Armonk, NY: IBM Corp. Descriptive statistics in our study were expressed as mean ± standard deviation (SD). The normal distribution of the data was assessed using the Kolmogorov-Smirnov test. Non-normally distributed data for parametric variables were analyzed using the non-parametric Mann-Whitney U test and Wilcoxon signed test, while normally distributed data were analyzed using the parametric independent samples *t*-test. Correlation analyses were performed using Pearson’s correlation for normally distributed data and Spearman’s correlation for non-normally distributed data. The statistical significance of the results was evaluated at *P* < 0.05 and a confidence level of 95%. Receiver operating characteristics (ROC) curve analysis was used to examine the sensitivity and specificity data for redox biomarkers.

## RESULTS

### Redox and Antioxidant System Biomarkers-Related Findings

The mean ± SD levels of redox biomarkers in coronary sinus samples collected preand post-CPB surgery for 28 patients (14 blood cardioplegia + 14 del Nido cardioplegia) and isolated valve surgery for 26 patients (12 blood cardioplegia + 14 del Nido cardioplegia) are presented in Table 2.

**Table 2:** Redox biomarkers and transcription factors related findings.

Biomarker groups	Parameters	Valve patient group (n = 26)		Coronary patient group (n = 28)	
		Del Nido	Blood	Del Nido	Blood
		Preand post-sinus blood	Preand post-sinus blood	Preand post-sinus blood	Preand post-sinus blood
Protein oxidation biomarkers	PCO (nmol/mg protein)	2.21 ± 0.93	2.19 ± 0.13	1.98 ± 0.25	2.03 ± 0.30
		1.90 ± 0.51^*^	2.12 ± 0.17^*^	1.94 ± 0.38	1.88 ± 0.26
	AOPP (µmol chloramine-T equivalent /L)	47.16 ± 7.97	38.82 ± 6.81	43.72 ± 7.68	40.14 ± 8.46
		47.50 ± 9.39	45.24 ± 10.15^*^	47.35 ± 8.96	47.19 ± 7.74^*^
Lipid peroxidation biomarker	L-OOH (µmol/L)	0.55 ± 0.18	0.53 ± 0.10	0.45 ± 0.16	0.49 ± 0.17
		0.50 ± 0.15	0.60 ± 0.16	0.49 ± 0.19	0.59 ± 0.30
Antioxidant capacity biomarkers	MnSOD levels (ng/mL)	14.7 ± 1.4	15.2 ± 1.30	12.78 ± 1.1	13.62 ± 1.14
		15.93 ± 1.43	16.1 ± 1.52	15.88 ± 0.5^*^	14.28 ± 0.63
	GPx activity (U/mg protein)	0.35 ± 0.04	0.36 ± 0.08	0.25 ± 0.04	0.26 ± 0.05
		0.33 ± 0.04	0.34 ± 0.05	0.27 ± 0.05	0.24 ± 0.08
	GPx levels (µU/mL)	137.18 ± 49.05	138.16 ± 44 .06	135.43 ± 35.9	134.42 ± 36.8
		143.40 ± 49.68	142.38 ± 48.56	145.18 ± 24.3	139.16 ± 24.6
	CAT activity (kU/µg protein)	304.10 ± 164.78	304.10 ± 164.78	304.10 ± 164.78	304.10 ± 164.78
		331.64 ± 181.89	331.64 ± 181.89	331.64 ± 181.89	331.64 ± 181.89
	CAT levels (KU/L)	138.5 ± 69.12	149 ± 74.16	136.9 ± 52	151.43 ± 43.2
		178.1 ± 64.76^*^	165.43 ± 70.12	135.7 ± 37.94	158.21 ± 38.65
Transcription factors	Nrf2 (ng/mL)	8.75 ± 4.06	7.98 ± 5.04	8.69 ± 2.46	9.12 ± 3.46
		9.52 ± 4.95	8.96 ± 5.45	9.36 ± 2.48	9.85 ± 3.26
	Keap1 (ng/L)	440.3 ± 140.9	532.7 ± 167.3	415.5 ± 108	432.9 ± 200.5
		488.5 ± 97.3	641.6 ± 203.9^*^	433.6 ± 102.3	537.0 ± 145.5^*^
	PGC-1α (ng/mL)	5.45 ± 2.3	5.05 ± 1.9	4.80 ± 1.58	4.67 ± 1.65
		5.62 ± 2.18	5.37 ± 2.2	5.50 ± 1.60^*^	5.54 ± 1.45

* =*vs.* pre-cardiopulmonary bypass sinus blood and *P* < 0.05 values are shown in bold

AOPP=advanced oxidation protein products; CAT=catalase; GPx=glutathione peroxidase; Keap1=Kelch-like ECH-associated protein 1; L-OOH=lipid hydroperoxides; MnSOD=manganese superoxide dismutase; Nrf2=nuclear factor erythroid 2-related factor 2; PCO=protein carbonyl group; PGC-1α=peroxisome proliferator-activated receptor-gamma coactivator-1 alpha

### Protein Oxidation-Related Findings

A significant decrease in PCO levels was observed in coronary sinus blood samples collected before and after CPB surgery in the valve patient group for both cardioplegia solutions. The post-CPB coronary sinus blood samples of valve patients with del Nido cardioplegia show that PCO levels were much lower in their post-CBP samples compared to the valve patient group with blood cardioplegia. In the coronary patient group, no significant variation was observed in coronary sinus blood samples collected before and after CPB surgery for both cardioplegia solutions. AOPP results of the post-CPB coronary sinus samples of both valve and coronary patients group revealed that AOPP levels were significantly higher in blood cardioplegia groups. No significant differences were found in cardioplegia groups in both preand post-CPB for other protein oxidation biomarkers (Table 2).

### Lipid Oxidation-Related Findings

The levels of L-OOH in coronary sinus blood samples collected before and after CPB surgery for both patient groups are shown in Table 2. No significant differences were found between preand post-coronary sinus blood samples for both cardioplegia solutions.

### Antioxidant Capacity Biomarkers-Related Findings

The levels of antioxidant capacity biomarkers in coronary sinus blood samples collected before and after CPB surgery for 28 patients and isolated valve surgery for 26 patients are shown in Table 2. MnSOD levels in coronary sinus blood samples post-CPB were significantly higher in the coronary patient group with del Nido compared to their pre-CPB values. Post-CPB CAT results of coronary sinus blood samples of valve patients indicate that CAT levels were significantly higher in the patient group with del Nido. No significant differences were found in cardioplegia groups in both preand post-CPB for other antioxidant capacity biomarkers (Table 2).

### Transcription Factors Related Findings

The levels of transcription factors in coronary sinus blood samples collected preand post-CPB for 28 patients and isolated valve surgery for 26 patients are shown in Table 2. No significant differences were found for Nrf2 between patient groups in coronary sinus blood samples collected preand post-CPB surgery periods. Keap1 results of the post-CPB coronary sinus samples of both valve and coronary patients group revealed that Keap1 levels were significantly higher in blood cardioplegia groups. PGC-1α levels in coronary sinus blood samples after CPB surgery were significantly higher in the coronary patient group with del Nido compared to their pre-CPB values.

### Evaluation of Diagnostic Sensitivity of Redox Biomarkers

**Fig. 1:**
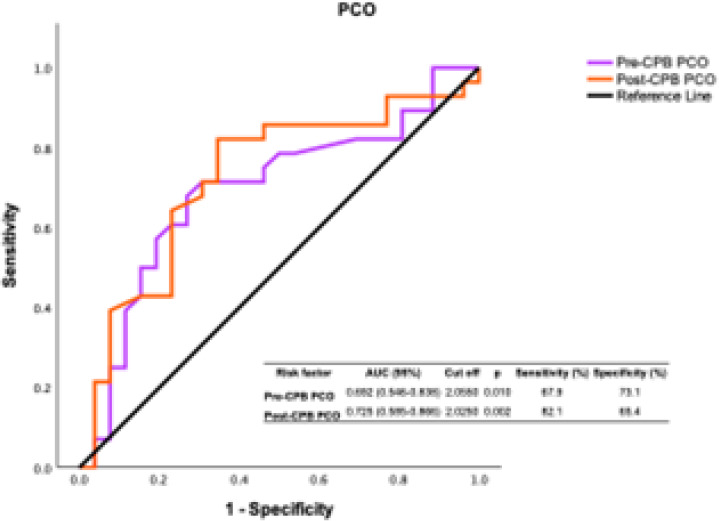
Receiver operating characteristic (ROC) analysis graph for protein carbonyl group (PCO) in preand post-cardiopulmonary bypass (CPB) patients. ROC curves were designed for PCO in both patient groups for all cardioplegias. AUC=area under the curve.

ROC curves were designed for PCO in both patient groups for all cardioplegias. According to the ROC analysis, PCO was found to be a diagnostically sensitive redox biomarker both preand post-CPB. This finding emphasizes the diagnostic utility of PCO groups in assessing the detrimental effects of CPB on redox status in blood samples collected from the coronary sinus preand post-CPB, which exhibit similar trends (Figure 1).

**Fig. 2:**
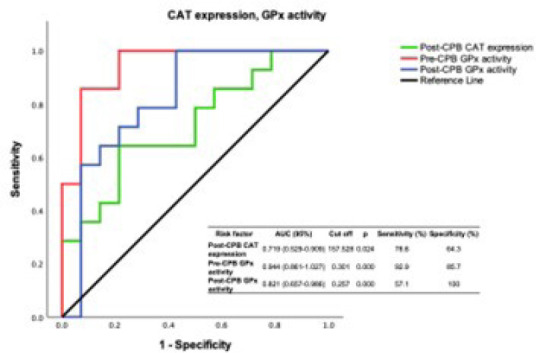
Receiver operating characteristic (ROC) analysis graph for glutathione peroxidase (GPx) activity and catalase (CAT) expression levels. ROC curves were designed for GPx and CAT biomarkers in both patient groups with del Nido cardioplegia. AUC=area under the curve; CPB=cardiopulmonary bypass.

**Fig. 3:**
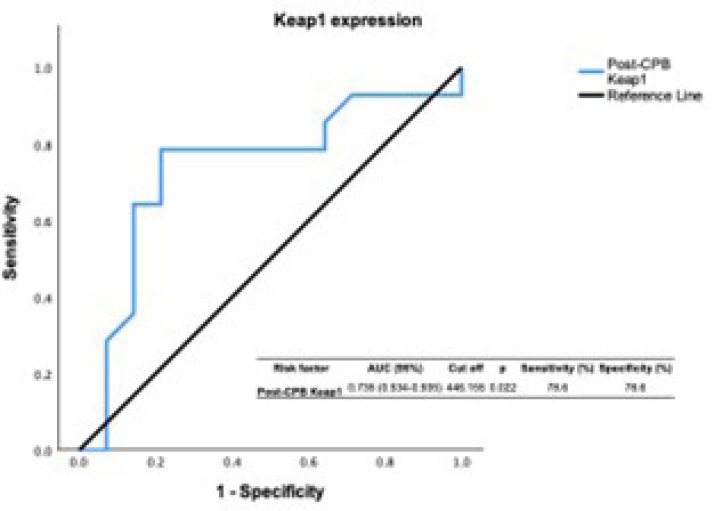
Receiver operating characteristic (ROC) analysis graph for post-cardiopulmonary bypass (CPB) Kelch-like ECH-associated protein 1 (Keap1) expression. ROC curves were designed for Keap1 in both patient groups with del Nido cardioplegia. AUC=area under the curve.

ROC plot graphics involve plotting pairs of sensitivity values of CPB patients with del Nido *vs.* (1-specificity) values of CPB patients with blood cardioplegia at all possible values for the decision threshold when sensitivity and specificity are calculated nonparametrically (Figures 2 and 3).

GPx activity was also found to be a redox-sensitive biomarker in preand post-CPB when assessing the antioxidant status of coronary sinus redox homeostasis. Like the results of ROC analysis of PCO groups, preand post-CPB. GPx activity showed excellent diagnostic performance among all statistically significant variations in antioxidant redox system biomarkers. The clinical significance of CAT expression as an antioxidant enzyme in removing the elevated L-OOH load in the coronary sinus circulation was shown by the ROC curve (Figure 2).

The ROC graph reinforced the value of Keap1 expression as an inhibitor biomarker, showing a similar trend to post-CPB GPx activity.

### Correlations

Correlation analysis results among various redox biomarkers and transcription factors in coronary sinus blood samples that were collected preand post-CPB with del Nido are given in Tables 3A and 3B.

**Table 3A:** Correlation analysis in the coronary patient group with del Nido cardioplegia.

		Post-CPB AOPP	Post-CPB MnSOD level	Post-CPB GPx activity	Post-CPB GPx level	Pre-CPB Keap1	Post-CPB Keap1	Post-CPB PGC-1α	CRP
Post-CPB PCO	r	0.641^*^	-	0.783^**^	-	-	0.612^*^	-	-
	*P*-value	0.014		0.001			0.020		
Post-CPB GPx activity	r	0.686^**^	-	-	-	-	-	-	-
	*P*-value	0.007							
Post-CPB CAT levels	r	-	0.764^**^	-	0.686^**^	-	-	0.717^**^	-
	*P*-value		0.001		0.007			0.004	
Post-CPB Keap1	r	-	-	-	-	0.740^**^	-	-	-0.600^*^
	*P*-value					0.002			0.023

* *P* < 0.05;

** *P* < 0.01

AOPP=advanced oxidation protein products; CAT=catalase; CPB=cardiopulmonary bypass; CRP=C-reactive protein; GPx=glutathione peroxidase; Keap1=Kelch-like ECH-associated protein 1; MnSOD=manganese superoxide dismutase; PCO=protein carbonyl group; PGC-1α=peroxisome proliferator-activated receptor-gamma coactivator-1 alpha

**Table 3B:** Correlation analysis in the valve patient group with del Nido cardioplegia.

		Pre-CPB MnSOD level	Post-CPB MnSOD level	Post-CPB GPx activity	Pre-CPB GPx level	Pre-CPB Nrf2	Post-CPB Nrf2	Pre-CPB PGC-1α	CRP
Pre-CPB GPx activity	r	-	-	0.673^**^	-	-	-	-	-
	*P*-value			0.008					
Pre-CPB CAT levels	r	0.725^**^	-	-	0.719^**^	0.548^*^	-	0.603^*^	-
	*P*-value	0.003			0.004	0.042		0.022	
Post-CPB CAT levels	r	-	0.675^**^	-	-	-	0.723^**^	-	-
	*P*-value		0.008				0.003		
Post-CPB Keap1	r	-	-	-	-	-	-	-	0.768^**^
	*P*-value								0.001

* *P* < 0.05;

** *P* < 0.01

CAT=catalase; CPB=cardiopulmonary bypass; CRP=C-reactive protein; GPx=glutathione peroxidase; Keap1=Kelch-like ECH-associated protein 1; MnSOD=manganese superoxide dismutase; Nrf2=nuclear factor erythroid 2-related factor 2; PGC-1α=peroxisome proliferator-activated receptor-gamma coactivator-1 alpha

### Routine Biochemical Parameters

The mean ± SD values of routine biochemical parameters in venous blood samples obtained post-CPB in 28 patients undergoing coronary artery bypass surgery and 26 patients undergoing isolated valve surgery are shown in Table 1. No significant difference was found between patient groups.

## DISCUSSION

The widely accepted paradigm regarding ROS - "increased oxidative stress, oxidative damage in macromolecules, and decreased or impaired function of cellular metabolic pathways" - has shifted towards investigating the physiological and pathophysiological importance of ROS-regulated redox signaling pathways^[18]^. ROS are considered hormetic and create cellular redox signals that facilitate transmission through adaptive proteins in the cardiovascular system tissue. Optimum levels of ROS formation regulate cell proliferation, differentiation, and excitation-contraction coupling in cardiomyocytes through redox signaling mechanisms^[19,20]^.

Surgery for isolated valve replacement and coronary artery bypass graft may cause ischemia and reperfusion damage in patients^[21,22]^. Of all cardiovascular surgery cases, valve surgery makes up about 10-20%^[23]^. Blood components are impacted both directly and indirectly by non-biological surfaces. During CPB, blood cells may be harmed by contact with non-endothelial surfaces. Non-pulsatile flow, blood interaction with non-endothelial surfaces, cross-clamping of cardiac blood flow, anesthetic medications, myocardial injury, complement system, and reperfusion are the primary causes of oxidative damage. Moreover, endothelial damage, the kallikrein cascade, neutrophils, catecholamines, the complement system, cytokines produced from active neutrophils, and endotoxin release contribute to the development of sterile inflammatory reactions^[24]^. Comparative research examining the effects of this redox dyshomeostasis on coronary sinus is not currently available in the literature. We thought redox status and signaling assessments were worth investigating in coronary sinus blood samples for the different cardioplegias.

In our study, the levels of widely accepted protein oxidation (PCO and AOPP) and redox signaling biomarkers in coronary sinus blood samples taken preand post-CPB were evaluated. In the pathogenesis of aortic valve disease associated with calcification, common risk factors such as ROS leading to endothelial damage, inflammation, hyperlipidemia, and low-density lipoprotein (LDL) oxidation play a crucial role, similar to their involvement in atherogenesis^[25]^. ROS and oxidized LDL lead to T lymphocytes and macrophage infiltration into the valve subendothelial tissue through a similar mechanism in the atherogenesis process.

In valve patient groups that were treated with both cardioplegia solutions, post-cross-clamping PCO levels were found to be significantly lower compared to pre-cross-clamping values. Both cardioplegias seem to be effectively protective against the detrimental consequences of protein oxidation products in our valve patient group. This finding might be explained by the presence of two different patient groups with similar pathogenesis and different surgical procedures that were followed. AOPPs are di-tyrosine-cross-linked and carbonyl-containing protein products and are reported to trigger inflammatory mechanisms involving cytokines (interleukin [IL]-6, IL-1), including macrophages, T lymphocytes, and mast cells, initiating the atherosclerosis process and serving as an early biomarker for atherogenesis^[26]^. In the coronary patient group with del Nido, the positive correlation between PCO and AOPP in the postoperative period suggests that a higher protein oxidation rate may be closely related to the PCO-reach oxidation product composition of AOPPs. A positive correlation was also found between post-CBP CAT and GPx levels in this group of patients. This positive correlation might indicate synergistic enzyme protein expression for post-CBP CAT and GPx to alleviate the detrimental effects of hydroperoxide-mediated protein oxidation.

For the levels of antioxidant system parameters except CAT and MnSOD, no significant difference was observed for del Nido cardioplegia groups. Increased antioxidant enzyme levels indicate the importance of these enzymes in eliminating the higher ROS load. Regulatory proteins of redox signaling did not exhibit significant variations except Keap1 and PGC-1α for both cardioplegias. Keap1 levels were found to be significantly higher in postoperative coronary sinus blood samples from both patient groups receiving blood cardioplegia. Increased Keap1 levels might be related to unchanged Nrf2 levels in our valve patient group, which was treated with both cardioplegia solutions. The Nrf2, which protects the cells from oxidative stress through natural antioxidant defenses, is thought to be a prime candidate for therapeutic targeting in the treatment of cardiovascular disease^[27]^. Natural products including baicalin, anthocyanin, diosmetin, and hesperidin, which are increasingly being observed as a potential source of Nrf2 activators with cardioprotective properties, may offer a new class of therapeutic pharmaceuticals for cardiovascular disease^[27]^. Keap1 inhibitors are also regarded as Nrf2 activators. According to recent studies, Keap1 is essential for proteostasis, mitochondrial homeostasis, cytoskeleton modulation, cell cycle progression, and Nrf2 regulation in the cardiovascular system. Beyond Nrf2 inactivation, the effect of these Nrf2 activators or Keap1 inhibitors on Keap1-mediated functions is still largely unclear^[28]^. The lack of difference in Nrf2 activation between patient groups is due to Nrf2 dysregulation in cardiovascular disease and the absence of Nrf2 modulators in CPB routines. We suggest that adding selective pharmacological agents to the CPB procedure to activate the Nrf2 signaling pathway could be beneficial to enhance its antioxidant effects. PGC-1α seems to exhibit myocardial protection in coronary patient group with del Nido in coronary patient group with del Nido.

ROC curves are an efficient way to display the relationship between sensitivity and specificity for continuous biomarkers. ROC analysis is a useful method for comparing redox biomarkers in coronary artery bypass graft patients^[29]^. ROC analysis results for oxidant systems indicate that PCO groups are a valuable redox biomarker with a sensitivity approaching 68% preand 82% post-CPB in both patient groups for all cardioplegias, with statistically advanced significance. This evaluation becomes even more evident at cutoff values corresponding to sensitivity values of 0.55-0.85. This suggests that the diagnostic value of PCO groups alone is high in assessing the oxidant effect of CPB on blood samples collected from the coronary sinus preand post-CPB. When examining the antioxidant side of the dynamic equilibrium of coronary sinus redox homeostasis, we found that GPx, in terms of enzyme activity, is a valuable redox biomarker after preand post-CPB patients with del Nido cardioplegia. Pre-GPx activity exhibits high diagnostic performance at sensitivity values of 0.86-1.00. The ROC graph of CAT expression shows a similar trend to post-GPx and reinforces its value as a diagnostic redox biomarker. The ROC graph, which displayed a pattern resembling post-CPB GPx activity, supported the usefulness of Keap1 expression as an inhibitor biomarker and reinforced the value of Keap1 expression as an inhibitor biomarker. Keap1 might play an inhibitory role in Nrf2-mediated GPx activity in the post-CBP period.

### Limitations

A major limitation of this study is the inclusion of only elective, isolated coronary artery bypass grafting and mostly AVR cases, leading to selection bias. Other limitations include reliance on assay kits for biochemical evaluation, a small sample size based on our grant support, and the absence of long-term postoperative follow-up due to time constraints.

## CONCLUSION

Current results indicate del Nido cardioplegia is effective in providing myocardial redox protection. Given these findings, it can be concluded that despite concerns about its use in clinical practice, del Nido cardioplegia could provide effective myocardial protection in patients undergoing open-heart surgery.

However, since the data is from selected elective surgeries, more is needed for urgent, emergent, and complex cases.

In the past, the widely accepted paradigm regarding ROS - "increased oxidative stress, oxidative damage in macromolecules, and decreased or impaired function of cellular metabolic pathways" - has shifted towards investigating the physiological and pathophysiological importance of ROS-regulated redox signaling pathways at present. The original physiological foundation of cardioplegia is rooted in the need to protect the myocardium against ROS-mediated damage during cardiac surgery by preventing ischemic injury, optimizing redox signaling, and reestablishing redox homeostasis. We believe that our work could contribute to the current literature and clinical practice on the cardioprotective signaling advantages of del Nido cardioplegia solutions and the reasons for their preference for surgery. The possible application of pharmacological agents with redox modulator properties intravenously or through cardioplegia during CBP is anticipated to regulate the impaired redox homeostasis. We strongly believe that novel pharmacological formulations, such as synthetic and natural redox modulatory substances, will soon enter the scene in cardioplegia formulations. In this respect, it has become an important reference for all resources to be produced in this field in the coming years.
